# The Habitats Humans Provide: Factors affecting the diversity and composition of arthropods in houses

**DOI:** 10.1038/s41598-017-15584-2

**Published:** 2017-11-10

**Authors:** Misha Leong, Matthew A. Bertone, Amy M. Savage, Keith M. Bayless, Robert R. Dunn, Michelle D. Trautwein

**Affiliations:** 1 Institute for Biodiversity Science and Sustainability, California Academy of Sciences, San Francisco, CA United States; 2 Department of Entomology and Plant Pathology, North Carolina State University, Raleigh, NC United States; 3 Department of Biology and Center for Computational and Integrative Biology, Rutgers, The State University of New Jersey, Camden, NJ United States; 4 Department of Applied Ecology and Keck Center for Behavioral Biology, North Carolina State University, Raleigh, NC United States; 5 Center for Macroecology, Evolution and Climate, Natural History Museum of Denmark, University of Copenhagen, Copenhagen, Denmark

## Abstract

The indoor biome is a novel habitat which recent studies have shown exhibit not only high microbial diversity, but also high arthropod diversity. Here, we analyze findings from a survey of 50 houses (southeastern USA) within the context of additional survey data concerning house and room features, along with resident behavior, to explore how arthropod diversity and community composition are influenced by physical aspects of rooms and their usage, as well as the lifestyles of human residents. We found that indoor arthropod diversity is strongly influenced by access to the outdoors and carpeted rooms hosted more types of arthropods than non-carpeted rooms. Arthropod communities were similar across most room types, but basements exhibited more unique community compositions. Resident behavior such as house tidiness, pesticide usage, and pet ownership showed no significant influence on arthropod community composition. Arthropod communities across all rooms in houses exhibit trophic structure—with both generalized predators and scavengers included in the most frequently found groups. These findings suggest that indoor arthropods serve as a connection to the outdoors, and that there is still much yet to be discovered about their impact on indoor health and the unique ecological dynamics within our homes.

## Introduction

Houses provide an enormous amount of habitat on a global scale^1^. Humans spend 90% of their time indoors^2^, providing ample opportunity for this environment and its species to affect mental^3^ and physical well-being^4^. Indoor environments host a wide diversity of life beyond human inhabitants and familiar indoor plant and pet species; there are also thousands of species of microbes (bacteria, archaea, and fungi) and hundreds of arthropod species^1,5^ found in houses. Despite the ubiquity of the indoor biome, we are just beginning to understand which species live where in homes and why, or for that matter, why some rooms in homes are biologically diverse and others less so. It is unclear if the rules that govern more natural ecosystems are similar to those that govern life and its regularities in the indoor environment where we spend most of our time.

Recent research has focused on characterizing bacterial and, to a lesser extent, fungal communities indoors. Indoor bacteria and fungi include a mix of outdoor and human-associated species^6,7^. Within a house, kitchens and bathrooms often have distinct microbial communities^8^, as do locations where dust settles, or even refrigerators when compared to cutting boards^9–11^. In addition, the composition of microbes in a particular home or room may be influenced by who uses that room, both in terms of the number and gender of people, but also the presence and identity of pets^9,12–14^.

Beyond bacteria and fungi, arthropods are the most diverse group of organisms found indoors. Because of their ubiquity and mobility, arthropods can serve as important agents of movement for microbiota^15,16^, but they also make up a diverse indoor community themselves^5^, with many species having functional consequences for homes, humans, or pets^17^. Relatively little work has been done to characterize the species diversity and indoor distribution patterns of arthropods in human homes, aside from extensive research on a handful of well-known pest species.

Recent studies have shown that the diversity of arthropods in homes extends well beyond recognized pest species^5,18^. The majority of the arthropod species found in houses are not primarily indoor inhabitants and have no known impact on humans. In fact, most species found indoors are simply filtered from the surrounding environment with the home acting in a similar manner to a “Malaise trap”^5,18^. On the other hand, the existence of a multi-billion-dollar pest industry suggests the prevalence of indoor arthropod species that do have direct impacts on humans (as structural, stored food products, medical, or simply nuisance pests). Thus, the indoor arthropod community reflects a spectrum of association with humans, from outdoor vagrants trapped indoors, to arthropods that thrive inside human dwellings as both non-pests and pests. Such has been the case for no less than four thousand years, and likely much longer^19^.

Here we examine how physical features within homes and residents’ lifestyles influence the diversity and composition of arthropods within houses. By analyzing data from our previous survey of arthropod diversity in 50 urban homes in Raleigh, NC, we first explore aspects of rooms that are associated with higher arthropod diversity. Then, by focusing specifically on arthropods that are prevalent and/or closely associated with the indoors, we test the hypothesis, suggested by studies of microbiota in modern homes and arthropods in ancient homes, that the rooms in homes differ predictably in their arthropod communities as a function of their environmental conditions and available resources. Finally, we consider whether the lifestyle of the inhabitants of homes affects the diversity of arthropods therein.

## Results and Discussion

### Overall arthropod diversity throughout house

We found that a variety of physical features had a significant effect on the number of morphospecies and families of arthropods found in particular rooms of houses (Table 1). Arthropod diversity in rooms tended to decrease the higher the floor level. However, common rooms were more diverse than other rooms, including basements, perhaps as a function of their size. Larger rooms have more area for arthropods to live and accumulate, but at least in our data set, the common rooms also had more doors and windows leading to the outside (common rooms on average had 1.4 doors leading to the outside and 5.7 windows compared to the average room’s 0.4 doors leading to the outside and 2.4 windows). Overall, as the number of doors and windows increased in a room, so too did the arthropod diversity (Table 1).

**Table 1:** Output of GLMM models for effect of room type, floor level, carpet, windows, and doors on arthropod diversity at the morphospecies (A) and family (B) levels. For categorical variables, common rooms, ground level, and carpeted rooms are set as baseline values, and models are fit with Poisson distributions. numMS = Number of morphospecies; numFam = number of families; roomType = whether room was an attic, basement, bathroom, bedroom, or common room; floorLevel3 = whether room was subterranean, ground level, or above ground level; numWindow = number of windows in room that led to the outside; numDoor = number of doors in room that led to the outside; carpet = whether or not room was carpeted.

Variable	Estimate	Std. Error	z value	Pr(>|z|)
A. AIC = 5143.6, BIC = 5193.1				
*Formula: numMS ~ roomType + floorLevel3 + numWindow + numDoor + carpet + (1|houseID)*				
(Intercept)	3.443703	0.087043	39.56	<0.001
Room Type (attic)	−1.159753	0.138444	−8.38	<0.001
Room Type (basement)	−1.061511	0.08444	−12.57	<0.001
Room Type (bathroom)	−1.113023	0.042995	−25.89	<0.001
Room Type (bedroom)	−0.685018	0.034758	−19.71	<0.001
Room Type (kitchen)	−0.731902	0.048708	−15.03	<0.001
Floor level (subterranean)	0.73038	0.078324	9.33	<0.001
Floor level (above ground level)	−0.44685	0.003749	10.63	<0.001
No Carpet	−0.235563	0.033441	−7.04	<0.001
# of Windows	0.039849	0.003749	10.63	<0.001
# of Doors	0.076138	0.013764	5.53	<0.001
B. AIC = 3746.0, BIC = 3795.5				
*Formula: numFam ~ roomType + floorLevel3 + numWindow + numDoor + carpet + (1|houseID)*				
(Intercept)	3.007044	0.080465	37.37	<0.001
Room Type (attic)	−1.169549	0.15963	−7.33	<0.001
Room Type (basement)	−0.863172	0.101692	−8.49	<0.001
Room Type (bathroom)	−1.011451	0.050215	−20.14	<0.001
Room Type (bedroom)	−0.581422	0.040989	−14.18	<0.001
Room Type (kitchen)	−0.601813	0.056649	−10.62	<0.001
Floor level (subterranean)	0.69076	0.095808	7.21	<0.001
Floor level (above ground level)	−0.308457	0.039926	−7.73	<0.001
No Carpet	−0.139677	0.039105	−3.57	<0.001
# of Windows	0.038071	0.004524	8.42	<0.001
# of Doors	0.05982	0.016774	3.57	<0.001

Our findings suggest that the more opportunities arthropods have to get into a particular room in a home, the more diverse the arthropods in that room are likely to be. Houses act as exceptional arthropod traps, with rooms that are more accessible to the outdoors providing points of entry for diverse arthropods that primarily live in the surrounding soil and vegetation. Thus indoor arthropod diversity is in large part a representation of arthropod diversity in the surrounding outdoors. Such a pattern influences not only the arthropods found indoors, but also the microbes likely to arrive on arthropods, as well as allergens to which denizens of homes are exposed. Indoor microbe diversity is known to follow a similar pattern. Window-ventilated rooms are more diverse than are those that are ventilated mechanically^20^. The idea that open rooms on ground floors come with increased uninvited biodiversity may not seem appealing, yet a growing body of evidence suggests that many of our chronic, modern diseases are associated with our failure to be exposed to biological diversity, particularly that of microbes, some that may be vectored by insects^21–23^. In this light, rooms with more kinds of arthropods may well be healthier rooms.

Another predictable pattern we recovered was that rooms with carpet had more arthropod diversity than did those with bare floors (Table 1). Similar patterns have been found with dust mite diversity and abundance^5,24^. However, while dust mites actually inhabit the carpet, it is possible that with its variable surface area and depth, carpet may also act as a trap for many arthropods, regardless of their normal habitat inside or outside of the home. Thus, carpeted rooms may both provide more habitat to host living arthropods and trap more dead arthropods.

### Community composition of arthropods across room types

To further understand the distribution patterns of specific arthropods that are frequently found indoors, we pruned our dataset to include only what we considered to be the core members of the indoor arthropod community by focusing on families that were frequently found between and within houses, resulting in 47 arthropod families (Table 2). The groups in this core indoor community represent a range of life history traits and taxa that in some cases live out their entire life cycles indoors [for example, silverfish (Zygentoma) and book lice (Liposcelididae)], while others live both in association with houses and the outdoors [for example, fruit/vinegar flies (Drosophilidae) and ladybugs (Coccinellidae)]. Interestingly, many indoor pest groups such as house centipedes (Scutigeridae), and pests like subterranean termites (Rhinotermitidae), fleas (Pulicidae), and bed bugs (Cimicidae) were not included in the core indoor community based on our filtering methods. While these taxa are closely associated with human houses, they are relatively rare in our data set^5^. They can be very abundant when present, or are conspicuous when present (house centipedes) but occupy a relatively small proportion of homes at any given time.

**Table 2:** Core indoor arthropod community and relative over/under representation in various rooms. Included arthropod families were found in at least 20 houses and had spread to at least 4 rooms in a single house. The 11 focal taxa that were analyzed further based on proportional occurrence data are indicated with an asterisk (*). First 3 columns after taxa names indicate the percentage of total houses (max 50), the percentage of total rooms (max 554), and percentage of houses when present in >4 rooms in that house. The last six columns are reflective of whether arthropod families are over/under represented in each room type. To calculate relative over/under representation in room types, we calculated the presence ratio for each arthropod family from the core community in each of the 6 room types, then standardized proportions between families by dividing the presence ratio for each room type by the sum of all room type presence ratios for a given family. If the relative proportion for a given room is >0.167, then it is overrepresented in that room type. Conversely, if the relative proportion is <0.167, it was underrepresented. A ‘U’ denotes that the family is underrepresented by at least half (<0.0835), and an ‘O’ denotes that the family is overrepresented in that room by at least double (>0.334).

Class	Order	Family	houses %	rooms %	>4%	Attic	Basement	Bathroom	Bedroom	Common	Kitchen
Entognatha (non-insect hexapods)	Collembola springtails	Entomobryidae slender springtails	78	19.1	18	U					
Insecta (true insects)	Zygentoma silverfish	Lepismatidae (silverfish)*	68	21.3	30		U				
	Orthoptera grasshoppers, crickets	Rhaphidophoridae (camel crickets)*	58	12.8	12	U	O	U	U		U
	Blattodea cockroaches	Blattidae (cockroaches)*	74	22.9	28						
	Hemiptera true bugs	Cicadellidae (leafhoppers)	82	16.2	16	U	U			O	
		Miridae (plant bugs)	44	6.5	2	U	U	U		O	
	Psocodea barklice, booklice, parasitic lice	Liposcelididae (booklice)*	98	37.4	52		U				
	Hymenoptera bees, wasps, ants	Braconidae (parasitoid wasps)	52	7.6	2	U	U			O	
		Eulophidae (chalcidoid wasps)	70	17	18	U	U			O	
		Formicidae (ants)	100	61.9	88						
		Platygastridae s.l. (parasitic wasps)	62	9.4	4	U	U			O	U
		Pteromalidae (Pteromalid wasps)	52	7.6	2	U	U	U			O
	Coleoptera beetles	Carabidae (ground beetles)	66	9.9	6		O	U			U
		Chrysomelidae (leaf beetles)	46	6	2	U	U	U		O	U
		Coccinellidae (ladybugs)	52	7.8	4		U	U		O	U
		Curculionidae (snout & bark beetles)	82	15.7	16	U				O	U
		Dermestidae (carpet beetles)*	100	57	78		U				
		Elateridae (click beetles)	74	14.6	14	U		U		O	
		Ptinidae (death watch & spider beetles)	60	12.1	10	U				O	
		Scarabaeidae (scarab beetles)	52	9.4	6			U			
		Silvanidae (flat bark beetles)	46	6.5	2	U		U		O	
		Staphylinidae (rove beetles)	54	7.2	2	U				O	
		Tenebrionidae (darkling beetles)	62	11.2	12			U		O	U
	Lepidoptera moths, butterflies	Noctuidae (owlet moths)	44	5.8	2	U				O	
		Pyralidae (snout & pantry moths)*	62	11	8	U	U	U			O
		Tineidae (clothes moths)*	60	8.8	4	U	U			O	
	Diptera flies	Calliphoridae (blow flies)	48	8.1	6	U		U		O	
		Cecidomyiidae (gall midges)	100	36.1	50	U	U				
		Ceratopogonidae (biting midges)	54	7.6	2	U	U			O	
		Chironomidae (non biting midges)	80	17	8	U	U			O	
		Culicidae (mosquitoes)*	82	19	18	U	U	U		O	
		Drosophilidae (fruit flies, vinegar flies)*	66	13.7	10	U	U	U		O	
		Muscidae (house flies)	44	6.3	2			U		O	
		Mycetophilidae (fungus gnats)	68	16.2	14	U				O	U
		Phoridae (scuttle flies)	82	17.3	20	U		U		O	
		Psychodidae (sand & moth flies)*	74	18.8	22	U				O	
		Scatopsidae (minute black scavenger flies)	50	6.9	2	U	U			O	
		Sciaridae (dark winged fungus gnats)	96	42.1	60	U					
		Tipulidae (large crane flies)	74	15.9	14	U				O	
Arachnida (arachnids)	Araneae spiders	Agelenidae (funnel weavers)	46	8.3	2	U	O	U			
		Gnaphosidae (ground spiders)	48	8.1	2	U				O	U
		Lycosidae (wolf spiders)	40	5.8	2			U	U	O	
		Pholcidae (cellar spiders)*	84	28	38	U					
		Salticidae (jumping spiders)	50	8.3	8	U	U	U		O	
		Theridiidae (cobweb spiders)	100	65.3	86						
Diplopoda (millipedes)	Polydesmida flat-backed millipedes	Paradoxosomatidae (millipedes)	58	12.6	14	U	O	U			
Malacostraca (crustaceans)	Isopoda woodlice, pillbugs, roly-polys	Armadillidiidae (pillbugs)	78	22	28	U	O				

**Figure 1:**
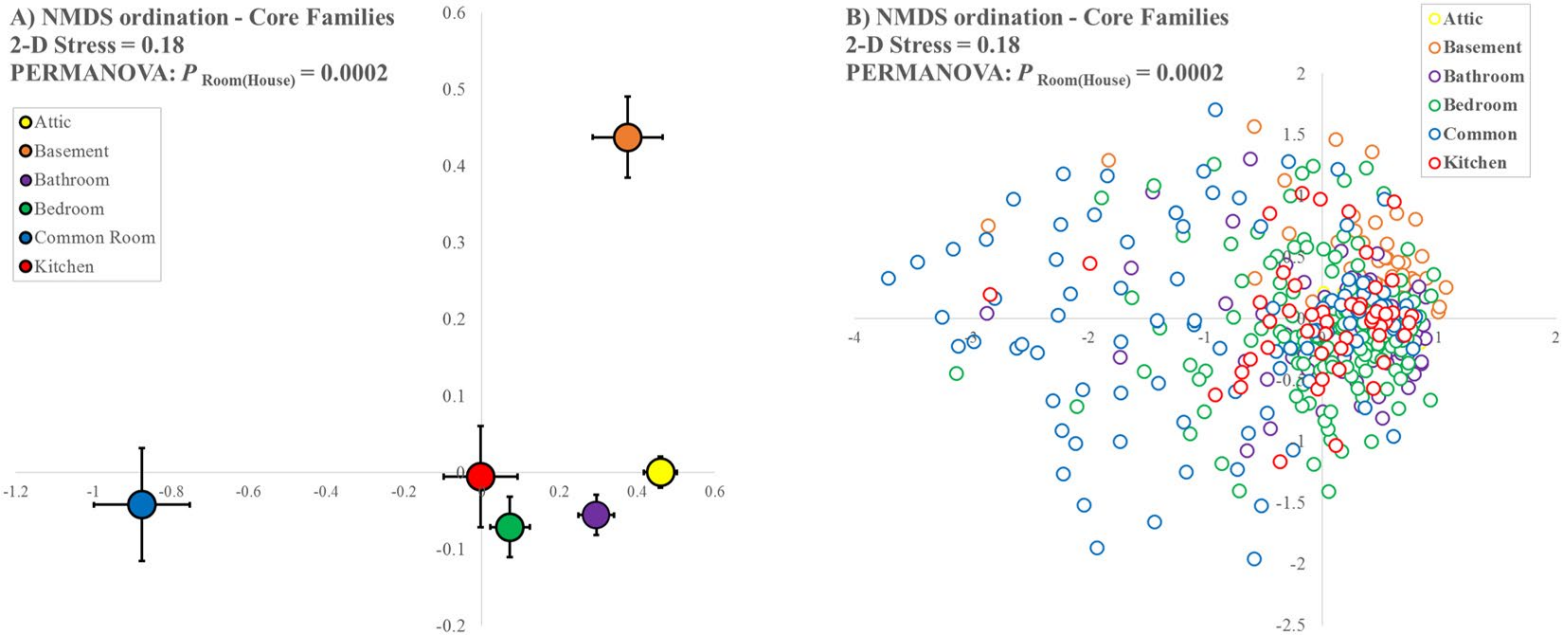
NMDS ordination of core arthropod families. NMDS plots were constructed using Manhattan distances of a presence-absence matrix at the level of individual rooms, 100 restarts, and a Type II Kruskal fit scheme. (**A**) Shows centroids ± 1SE for each room type, while (**B**) shows raw values for each room that was sampled. PERMANOVA analysis was conducted with 9,999 iterations.

We used our core community dataset to examine whether the composition of arthropod communities varied according to room type. We found that room type does explain some of the variation in arthropod communities within houses (PERMANOVA: P_Room(House)_ < 0.001), but only a modest amount (Fig. 1). Basements and common areas appear to host arthropod communities with significantly different compositions relative to those found in the more homogenous communities of bathrooms, kitchens, and bedrooms.

**Figure 2:**
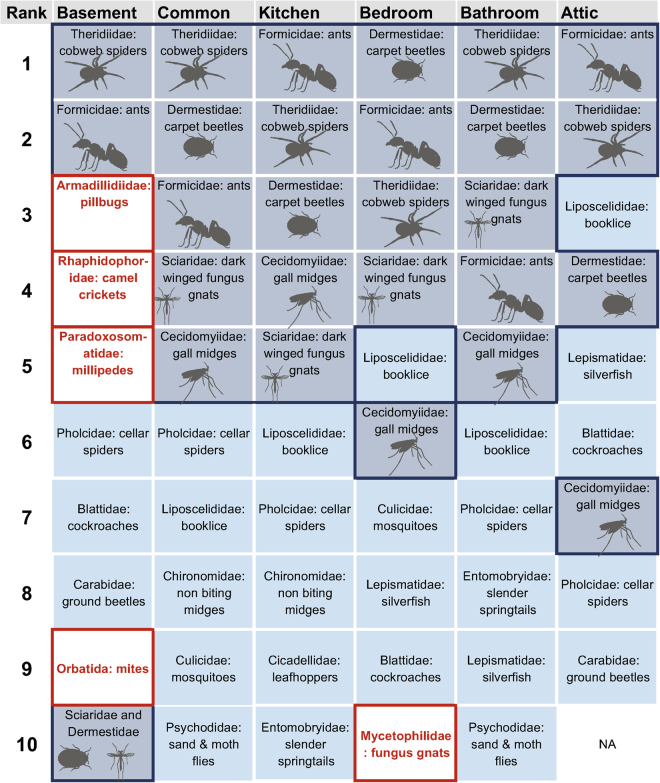
Rank occurrence of families by room type from most commonly collected to least. Highlighted in gray are the 5 most frequently found families across most room types, excluding basements and attics. Highlighted in red are families that occur in only one room type. Note that when more than one family share the same rank for a particular room, they are presented in the same box.

**Figure 3:**
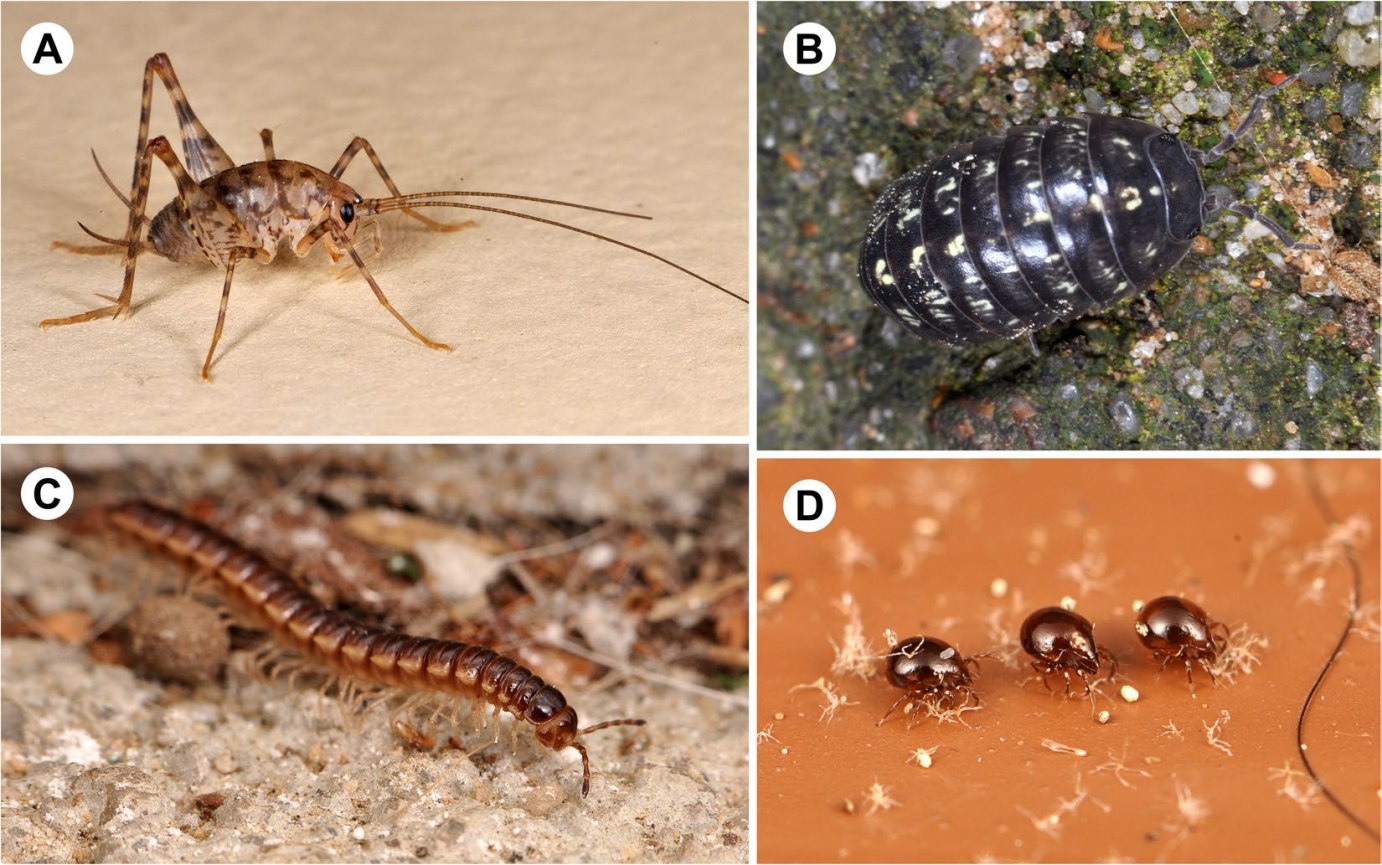
Images of most commonly collected famlies in basements. Photos by MAB. Basements had a unique set of arthropod families. (**A**) camel crickets (Rhaphidophoridae), (**B**) pill bugs (Armadillidae), (**C**) Millipedes (Paradoxosomatidae), (**D**) Moss mites (Oribatida).

To further explore the composition of these variable arthropod communities by room type, we considered the rank abundance of families for each room type and also calculated a metric of family under/over representation by room type. Based on these different methods, we found the basement/crawl space arthropod community to be most unique in terms of its composition (Table 2, Figs 2 and 3). Basements are cave-like in terms of their climate and sparse resources. They are often entirely subterranean, have low light levels, high humidity, and are not strongly influenced by human foods sources or the food generated by human bodies (e.g., skin cells, hairs, etc.). We find that basement communities include a greater diversity of arthropod orders than do other rooms—including more types of spiders, mites, millipedes, and isopods (crustaceans). Basements include an overrepresentation of cave-dwelling scavengers and predators—such as camel crickets (Rhaphidophoridae), millipedes (Paradoxosomatidae), terrestrial isopods (Armadillidiidae), and ground beetles (Carabidae) (Table 2). Groups underrepresented in basements primarily included flying insects—flies, moths, and wasps, along with jumping spiders that rely on their strong visual skills for hunting. Surprisingly, silverfish (Lepismatidae) and booklice (Liposcelididae) were also underrepresented in basements where they would be expected to thrive based on the damp environment they are thought to prefer.

Several groups of arthropods commonly considered to be room specific, were instead similarly represented across all room types (Table 2). Ants (Formicidae) and cockroaches (Blattidae) are often thought of as kitchen pests, but were found fairly evenly across room types. Pantry moths (Pyralidae) were the only family of known kitchen pests found to be overrepresented in kitchens. Despite their common name, cellar spiders (Pholcidae) were also collected frequently across room types and were missing from the ten most frequently collected families in just bedrooms and (Fig. 2). Moth/drain flies (Psychodidae) are pests associated with drains and pipes in bathrooms and were in fact frequently collected in bathrooms, as well as in common rooms (Fig. 2).

**Figure 4:**
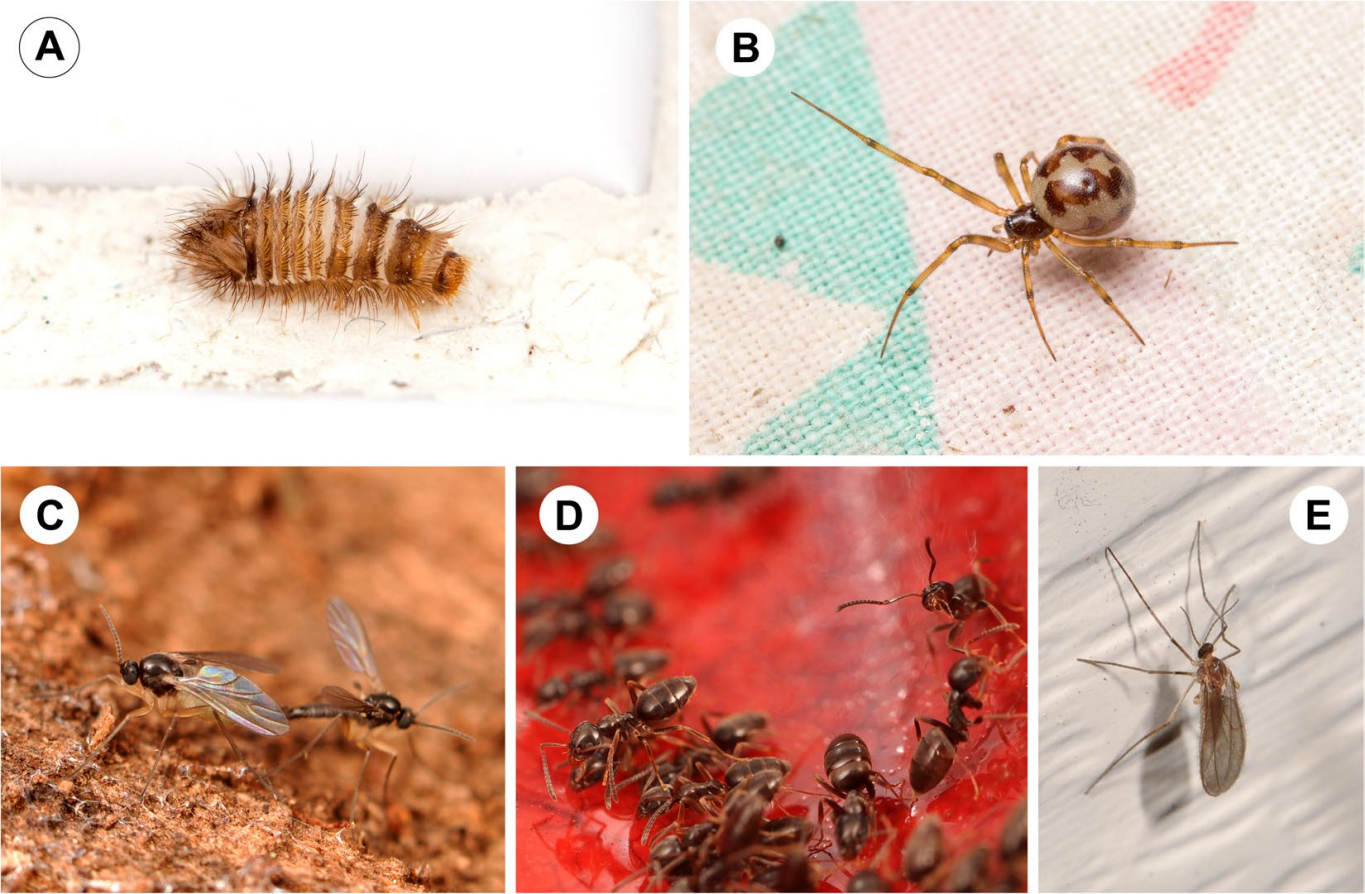
Images of most commonly collected families overall. Photos by MAB. (**A**) Carpet Beetles (Dermestidae), (**B**) Cobweb spiders (Theridiidae), (**C**) Dark winged fungus gnats (Sciaridae), (**D**) Ants (Formicidae), and (**E**) gall midges (Cecidomyiidae) were among the top 5 families for the majority of rooms.

We found that there was a great deal of homogeneity in the most frequently recovered arthropod families across all rooms—except for the attic and the basement. Ants (Formicidae), cobweb spiders (Theridiidae), carpet beetles (Dermestidae), dark winged fungus gnats (Sciaridae), and gall midges (Cecidomyiidae) were almost always the five most frequently found families in bedrooms, bathrooms, common rooms and kitchens (although booklice managed to penetrate this core group for bedrooms) (Figs 2 and 4). Arthropod communities across all rooms exhibited trophic structure—including diverse, generalized predators (spiders, ground beetles), house-associated scavengers and fungus feeders (ants, carpet beetles, book lice, drain flies, silverfish), and transients from outdoors (non-feeding flies, leafhoppers, springtails). Indoor arthropod communities are made up of predators and prey that are close human associates that thrive within homes, as well as those that primarily live outdoors, but strayed indoors.

### Community composition and diversity of arthropods associated with lifestyle factors

**Figure 5:**
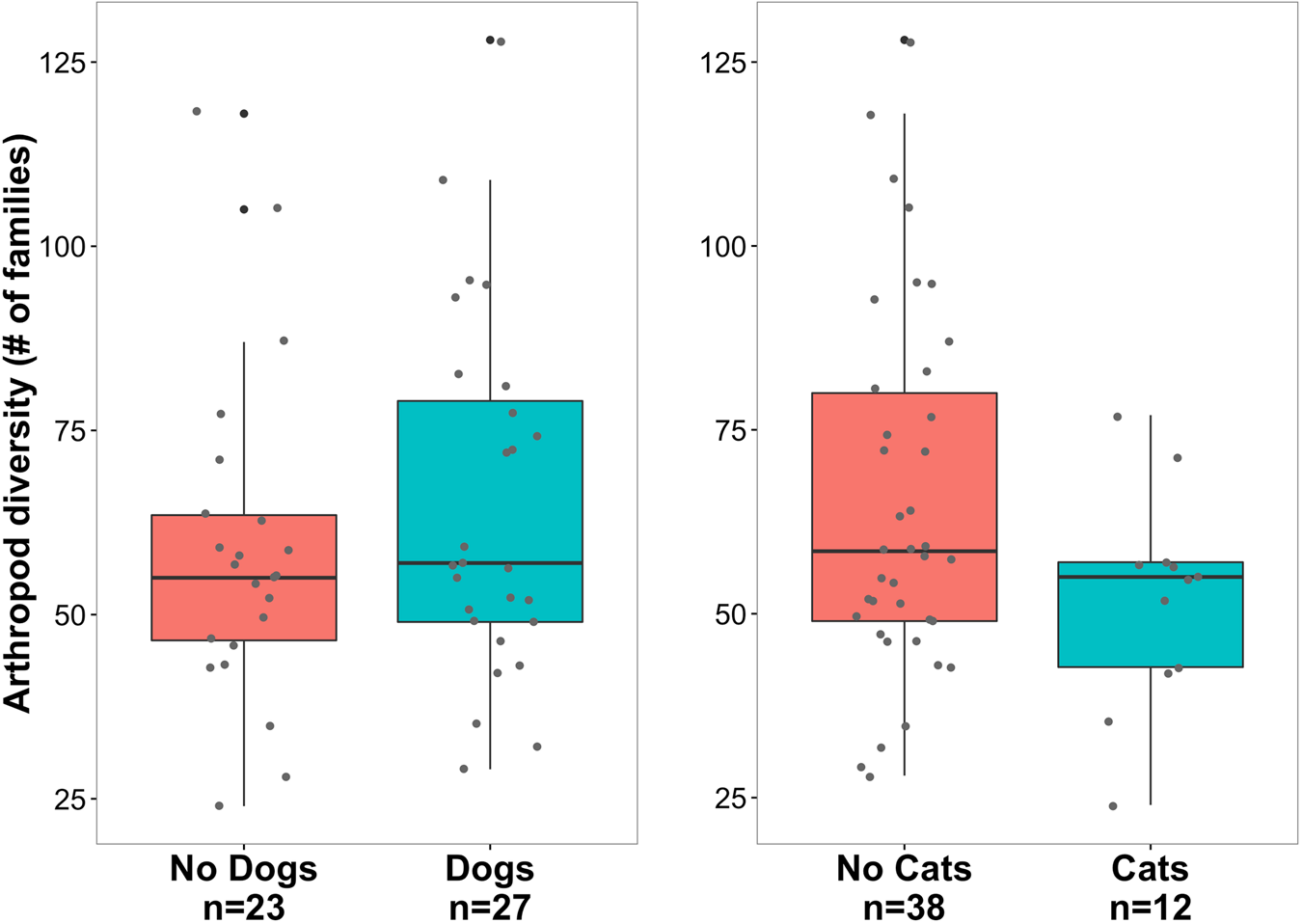
Mann-Whitney U test comparisons between arthropod family richness and presence of pet dogs or cats. No statistically significant differences were detected, but houses with dogs trended toward higher arthropod diversity, while houses with cats trended toward less.

When we considered lifestyle characteristics of residents within particular homes, we found that the diversity and the composition of the core arthropod community did not significantly vary between houses based on presence of cats or dogs, number of houseplants, pesticide usage, or relative levels of clutter and dust accumulation. However this could be due to sample size—a study of dust samples from 1462 houses across the United States found greater arthropod diversity in houses with pets^25^. Although we did not find a significant difference in diversity for pets in our study, there was a trend toward greater arthropod diversity in houses with dogs and, intriguingly, reduced arthropod diversity in houses with cats (Fig. 5). While our data does not differentiate between outdoor and indoor house cats, reduced arthropod diversity could be due to cats actively hunting arthropods indoors.

Out of the 47 core community arthropod families, we focused more closely on 11 of these for which the biology of their association with houses has been well-described (Table 2). These families include silverfish, camel crickets, cockroaches, booklice, carpet beetles, pantry moths, clothes moths, mosquitoes, fruit flies, moth flies, and cellar spiders—and together they constitute a range of higher-level taxonomic diversity as well as life history traits. The only significant relationship we found with house variables was that more cluttered houses had slightly higher proportional occurrence (the proportion of rooms in a house present) of cellar spiders (Pholcidae) (Kruskal-Wallis *x* ^2^ = 6.389, p = 0.04). None of the other interior variables (presence of cats or dogs, number of houseplants, pesticide usage, or relative levels of dust accumulation) were associated with the abundance of a particular taxon. Cellar spiders are relatively large arthropods, so perhaps they would be less likely to accumulate in tidier houses, and cluttered areas may offer more structures on which they can build their webs.

Together, these results suggest that human behavior may have little impact on the diversity and composition of the types of arthropods found indoors. Arthropods in human houses are an inevitable part of life on Earth and more reflective of the conditions outside homes than the decisions made inside.

## Conclusion

**Figure 6:**
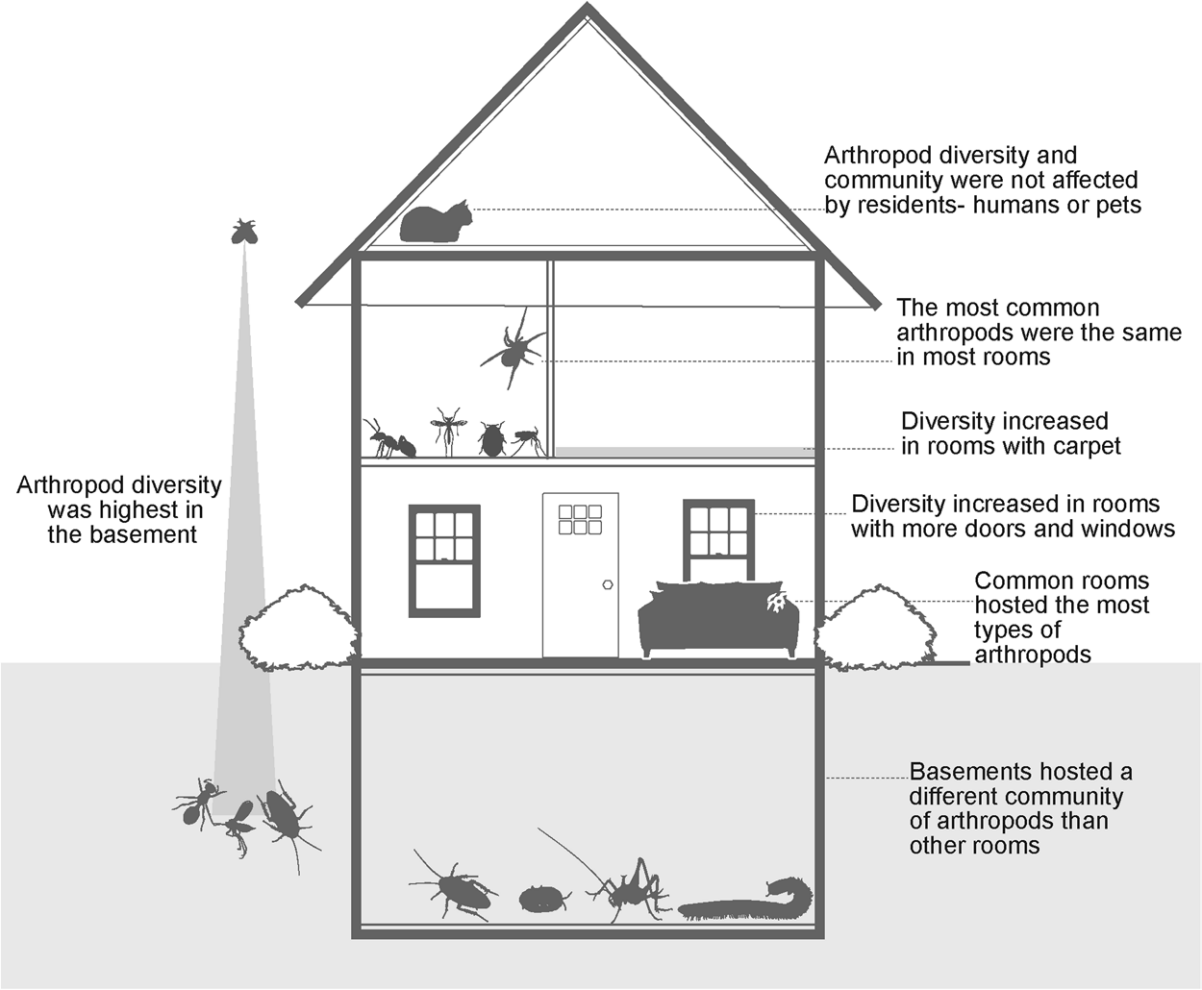
Summary of findings.

Our work here highlights the variability of arthropod responses to the indoor ecosystem and builds upon an increasing number of studies that characterize non-human life in the built environment^1,5,6,8,9,12,25^. Human houses, containing many microhabitats, host a gradient of biodiversity—including microbial and arthropod species associated with the outdoors, as well as groups that have adapted to live in close association with humans and their houses^1,5,9^. Arthropod diversity indoors, largely made up of outdoor vagrants and infrequently collected families, indicates that houses are in part like Malaise traps that passively filter arthropods from the surrounding landscape; this is suggested by the fact that we find higher arthropod diversity in association with factors related to house permeability (room level, number of windows and doors; Fig. 6).

When we consider only arthropod groups that live in close association with humans, we find evidence of community structuring in specific room types—particularly in basements versus above ground rooms. Indoor arthropod ecology can be considered similar to island ecology, in that dispersal limited species sometimes reproduce and spread within a house once it is colonized. These core indoor groups likely have a trophic system that relies not only on other house associates, but also on the regular influx of outdoor vagrants. In addition, we find that indoor arthropod diversity is consistent across a diversity of lifestyle choices by human inhabitants; cleaning, pesticide use, hosting of indoor plants and pets have no substantially significant effect on the diversity and make-up of indoor arthropods—perhaps the diversity of arthropods in the surrounding outdoor environment is the primary driver of their diversity indoors.

A growing understanding of the complexity of these community dynamics may lead to better pest management, reduction of unnecessary public health hazards, and a shift in cultural acceptance of the nature that surrounds us. Additionally, new insights on the biodiversity of the indoor biome can be beneficial to archaeoentomologists reconstructing human history through the use of environmental clues provided by insect remains found at formerly human-inhabited archeological sites. However, it is challenging to extrapolate meaning from these historical remains, when present-day dynamics are still poorly understood^26^.

As humans, particularly in western societies, we view ourselves as distinct from the natural world. Our species has been building houses to shelter itself from outdoor exposure for about 20,000 years^27^. Yet our houses, however isolated they may seem, actually host a range of biodiversity that keeps us connected to the natural world. This influx of outdoor biota, though not always welcomed, may contribute to our health and immunity in ways yet to be fully understood.

## Methods

### Arthropod dataset

We used data drawn from collections of arthropods in fifty homes within a 65 km radius of central Raleigh, North Carolina, U.S.A. Both living and dead arthropod specimens on all visible surfaces were exhaustively sampled in every room in the house through hand-collecting by trained entomologists (further details^5^). Specimens were identified to the family level in most cases (with provisional IDs of genera and species, where possible). Ultimately, this dataset includes over 10,000 specimens that comprise 304 unique arthropod families. Each home had 24–128 (mean = 61.84; s.d. = 23.24) distinct arthropod families and 32–211 (mean = 93.14; s.d. = 42.34) conservatively estimated morphospecies per house^5^.

A map of house locations can be found in Bertone *et al*.^5^. Houses varied in their distance from one another, ranging from the closest pair being separated by only 37 meters, and the furthest pair separated by 71.3 km (mean = 19.84 km; median = 18.86 km). We did not detect any spatial autocorrelation of arthropod diversity by house location (Moran’s I = 0.007, p = 0.638). Additionally, houses in the study ranged from seven to 94 years old (mean = 41.35; median = 30.5). House age was not determined to be an important factor when modelling house level arthropod diversity in Leong *et al*.^18^.

### Overall arthropod diversity throughout house

During the course of sampling fifty homes, 554 rooms were investigated, and we calculated the total number of morphospecies and families collected in each room. We used 531 of these rooms in the analyses below, as this subset could be categorized into attics, basements, bathrooms, bedrooms, common rooms, and kitchens (Table 3); rooms not conforming to one of the categories were classified as “other” and excluded from formal analyses. Floor levels ranged from zero (basements and crawl spaces) to four, but because very few houses had this many floor levels, we simplified this to subterranean (0), ground level (1), and above ground level (2). Due to the diversity of floor types, rooms were categorized as carpeted or bare.

**Table 3:** Room types, their definitions, and associated data. Columns denote how many of this room type were included in the dataset (N); the house levels a room type was associated with [we simplified this to be subterranean (0), ground level (1), and above ground level (2)]; and the number of houses out of 50 possible that this room type was found in. Some houses did have multiple underground rooms, which resulted in more basements than houses. We did not collect in garages, and in order to qualify as a “basement” a room had to be subterranean. We did not find any arthropods in one of the houses’ bathrooms, so those were not included in the dataset.

Room Type	N	Range of house levels	Homes with room type
**Attics**	32	2	31
Basements (including finished, unfinished, & crawl spaces)	53	0	45
Bathrooms (including bathrooms & laundry rooms)	140	1–2	49
Bedrooms (including bedrooms, offices, & libraries)	159	1–2	50
Common Rooms (including living rooms, dining rooms, & attached hallways)	97	0–2	50
Kitchens (including kitchens & pantries)	50	1	50
**Total**	**531**	**0–2**	**50**

We tested for diversity patterns related to each room’s categorical (room type, floor level, and floor type), and continuous variables (number of windows and doors leading to the outside) with generalized linear mixed models using the R package *lme4* ^28^. Response variables were the number of morphospecies and families collected in each room, with house designated as a random effect. The common room type was the model baseline for the categorical variable of room type, and the ground floor level as the baseline for floor level. The models were fit with Poisson distributions.

### Community composition

We sought to explore the community composition of the indoor arthropod community—specifically whether arthropods, like microbes, are partitioned by room type. To see which arthropod taxa were most closely associated with certain room types, we generated ranked lists of the 10 most common families for each room type to qualitatively compare between rooms.

Next, because almost half of all collected arthropod families were collected from fewer than 10% of homes—66 of which were found in just one of 50 homes (indicating they are not truly representative of frequent indoor dwellers)—we pruned our arthropod dataset to include only key groups that are reliably associated with the indoor environment. To be included as a core member of the indoor arthropod community, arthropod families had to be found in at least 20 of the 50 houses, which was where we found a natural break in the data. We then assumed that once present in a house, members of the core indoor community could survive, reproduce and spread through multiple rooms within a particular house. Therefore, from within this subset of commonly collected arthropods, we included in the core community all arthropod families that were found in at least 4 rooms in a single house, which was half the number of rooms in the average house.

We then explored whether these core community taxa were evenly distributed between rooms. To examine the over/under representation of arthropod taxa within a room type, we calculated the presence-absence ratio for each arthropod family from the core community in each of the 6 room types. For example, if an arthropod family was present in 20 attics, its presence ratio would be 0.625 because there were 32 possible attics it could be collected in, whereas if it was collected in 20 bathrooms, its ratio would be 0.14 because there were 140 possible bathrooms in which it could have been collected. We then standardized these proportions between families, to make it easier to compare highly abundant families against those that were collected less frequently. These relative proportions were calculated by dividing the presence ratio for each room type by the sum of all room type presence ratios for a given family. If an arthropod family was equally likely to be collected in a bathroom as it was a basement, because there are 6 room types being evaluated, the relative proportion for each room type would be $1/6$, or 0.167. Therefore, if the relative proportion for a given room is >0.167, then it was overrepresented in that room type. Conversely, if the relative proportion is <0.167, it was underrepresented.

Finally, we examined how the arthropod community was influenced by characteristics that varied within rooms and among houses by constructing a presence/absence matrix for the core families by all rooms. We used this matrix (+1 to control for multiple zeros) to make a distance matrix using Manhattan (city-block) distances. Next, we conducted PERMANOVA (Permutational Multivariate Analysis of Variance)^29^ analysis with 9,999 iterations. We visualized community composition using NMDS (Non-Metric multi-Dimensional Scaling) with 100 restarts and a Type II Kruskal fit scheme. We applied a stress cut-off of 0.20; if stress was >0.20, we considered the NMDS plot to be unreliable.

For each home in the study, we gave residents a survey, which included information about factors that may have influenced arthropods in the home (S1 dataset). The survey included among-homes factors that were separated into two groups: (i) human behaviors, which included pesticide use, clutter, and filth; and (ii) other organisms in the home, which included the number of cats, the number of dogs, and the number of houseplants in each house. Clutter and filth were based on a scale of 1–5, and determined by consistent members of our team of entomologists who visited every house and assessed them relative to one another. We combined these data with the aforementioned within-room factors, which were: floor level, number of windows, number of doors, and flooring.

We applied a stepwise approach to assess the effects of the room-level and home-level factors on arthropod composition. We first tested for all main effects, 2 and 3-way interactions; we then removed any non-significant interaction terms (*P* > 0.05). We again used Manhattan distances and 9,999 iterations for these PERMANOVAs.

### Focal taxa

We delved even further into our core community list to select a subset of families for which the biology of their association with houses has been well-described and where reproduction within the house is possible (see Results and discussion). We created a proportional occurrence metric for each of these families by calculating the proportion of rooms within a house in which they were present. Using these proportional occurrence metrics as response variables, we then tested how they varied with the indoor survey house-level factors using Kruskal-Wallis tests.

### Data availability

The arthropod dataset is available as a supplemental file in Bertone *et al*.^5^ doi:10.7717/peerj.1582/supp-2.. Additional survey data concerning house and room features, along with resident behavior can be found as a supplemental file with this paper (S1 dataset).

## Electronic supplementary material

Dataset 1
